# A mild form of dermatomyositis as a prodromal sign of lung adenocarcinoma: a case report

**DOI:** 10.1186/s13256-016-0816-8

**Published:** 2016-02-06

**Authors:** Eleni Papakonstantinou, Alexander Kapp, Ulrike Raap

**Affiliations:** Department of Dermatology and Allergy, Hannover Medical School, Carl-Neuberg-Str. 1, 30625 Hannover, Germany

**Keywords:** Dermatomyositis, Lung adenocarcinoma, Malignancy, Nail changes

## Abstract

**Background:**

Dermatomyositis is an idiopathic connective tissue disease characterized by specific cutaneous findings and inflammatory lesions in the muscle biopsy. An association between dermatomyositis and malignancy, including breast, ovarian, lung and colon cancer was recognized many years ago, with an incidence of malignancy in approximately 20 % of cases. Dermatomyositis is hypothesized to be an autoimmune reaction against factors or hormones secreted by the tumor; however, the exact autoimmune mechanism of the disease pathogenesis remains unknown.

**Case presentation:**

Here we report a case of a woman with dermatomyositis who was diagnosed with lung adenocarcinoma in the setting of weight loss, progressive fatigue and muscle weakness. A 43-year-old Caucasian woman was referred to our hospital by her physician for suspected contact dermatitis since she described mild itching sensations in her arms and legs as her major symptom. A physical examination revealed erythematous papular lesions over her metacarpophalangeal and proximal interphalangeal joints together with a periungual involvement with redness, hyperkeratosis and capillary telangiectasia along the distal nailfolds on her hands. She was unaware of these features and they did not seem to bother her. A thorough examination of her medical history, however, revealed more symptoms. Pain and weakness in the muscles of her proximal extremities and neck flexor muscles led to difficulty in raising her arms and climbing stairs. At the same time she experienced swallowing difficulties and reported an uncharacteristic weight loss of 10 kg in the last 3 months. The results of laboratory tests showed increased values of serum creatine kinase and myoglobin. An electromyogram, a skin biopsy and a muscle biopsy confirmed the diagnosis of dermatomyositis. A computed tomography of her thorax showed a nodular mass in the upper lobe of her right lung. A histological examination of the lung biopsy showed an adenocarcinoma of moderate differentiation. She was diagnosed with paraneoplastic dermatomyositis as the first sign of a lung adenocarcinoma.

**Conclusions:**

Our case report highlights the importance of a thorough search for underlying malignancy in patients with dermatomyositis even if dermatomyositis has a mild appearance or a discrete skin manifestation.

## Background

Dermatomyositis is an idiopathic inflammatory disease that is caused by an autoimmune reaction against skin and muscles. Viral infections, drugs or malignancy are the most commonly suggested trigger factors that may lead to an immune response. Dermatomyositis has typical cutaneous features and can appear with or without an inflammatory myositis.

The most common skin manifestations are an erythema over the face, neck, and upper trunk known as “neckline V sign” and slightly elevated erythematous papules located on the metacarpophalangeal and proximal interphalangeal joints, called “Gottron’s papules” which are found in 60 to 80 % of patients. A purple-red discoloration of the upper eyelids with or without associated eyelid edema may be present. Nail changes such as capillary telangiectasia along the distal nailfold, periungual erythema, and hyperkeratosis are common and, although usually ignored by the patients, can be a prodromal sign of dermatomyositis. More rarely calcinosis or hyperkeratotic lesions on the fingers known as “Mechanic’s hand” appear.

Other cutaneous symptoms include photosensitivity and pruritus [1]. The muscle symptoms consist of mild to severe weakness of the proximal muscles, followed by pain in one third of cases. These patients have difficulties raising their arms or climbing stairs. Muscle cramps and diffuse unexplained fatigue may be present as well. Dysphagia, weight loss, or dyspnea may appear over time and are usually signs of disease progression or underlying malignancy. The clinical presentation of dermatomyositis and the severity of clinical symptoms vary from patient to patient. There is no specific sequence of the symptoms and not all of them have to be present in order for the diagnosis to be verified. Dermatomyositis may also have atypical manifestations or appear in an incomplete form.

## Case presentation

**Fig. 1:**
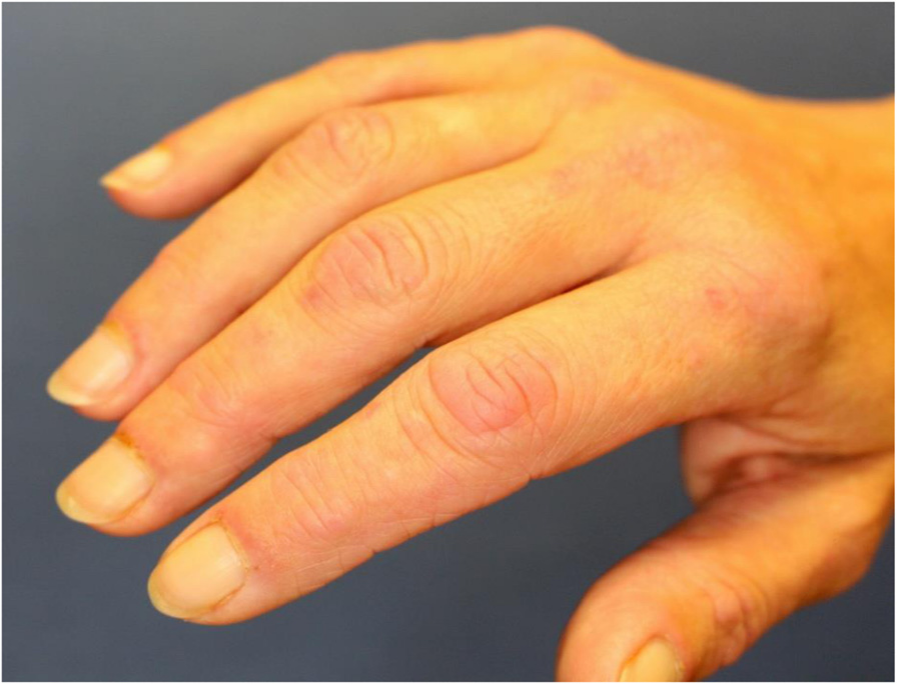
Erythematous papular lesions over the metacarpophalangeal and proximal interphalangeal joints (Gottron’s papules), as well as periungual erythema and hyperkeratosis along the distal nailfolds on both hands of our patient

**Fig. 2:**
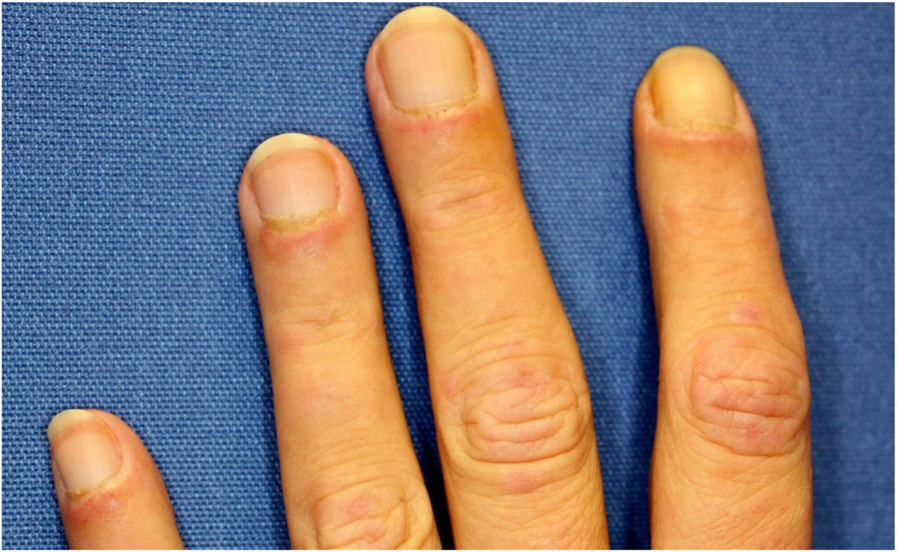
Periungual erythema and capillary telangiectasia along the distal nailfolds on both hands of our patient

A 43-year-old Caucasian woman presented to our clinic with a mild itching sensation in her arms and legs. She was referred by her physician for contact eczema. A physical examination revealed erythematous papular lesions over her metacarpophalangeal and proximal interphalangeal joints (Fig. 1) together with a periungual involvement with redness, hyperkeratosis, and capillary telangiectasia along the distal nailfolds on both her hands (Fig. 2). No other skin findings such as heliotrope rash or erythema on the extensor surface of her extremity joints were present.

She complained about pain and weakness in the muscles of her proximal extremities and neck flexor muscles with difficulty raising her arms and climbing stairs. At the same time she experienced swallowing difficulties and reported an uncharacteristic weight loss of 10 kg in the last 3 months. She had a 30 pack-year history of cigarette smoking with persistent nicotine use at the time of presentation in our clinic. A neurological examination showed symmetrical mild proximal muscle weakness. The rest of her physical examination including auscultation of her lungs, body temperature and lymph nodes status was normal.

**Fig. 3:**
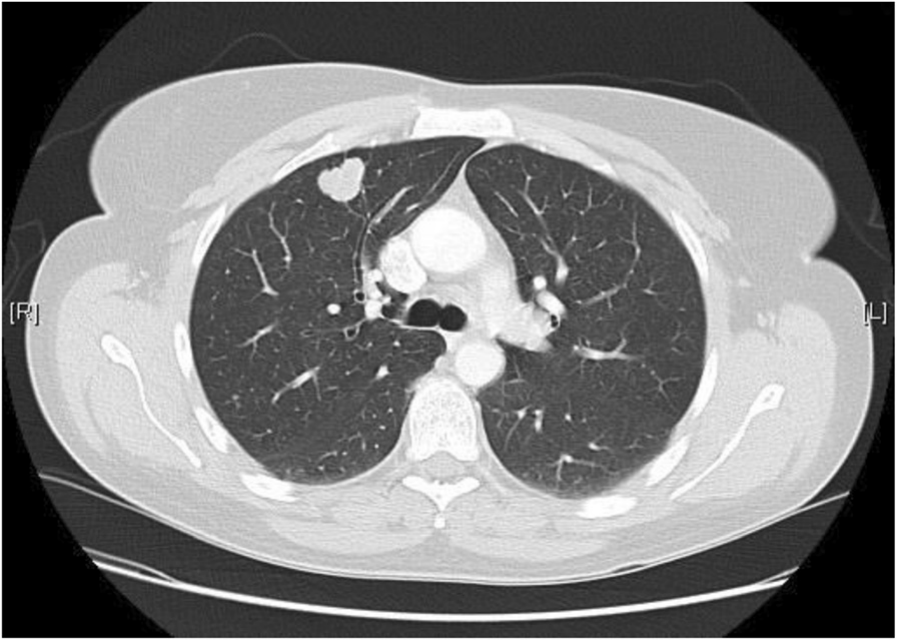
Computed tomography of the patient’s chest showing a speculated nodule in the upper field of her right lung

The laboratory findings revealed elevated serum levels of myoglobin 397 μg/l (normal range 25 to 58), creatine phosphokinase (CK) 881 IU/L (normal range 0 to 145 IU/L) and aldolase 11.8 U/l (normal range up to 7.6 U/l). Her liver enzymes were slightly elevated as were aspartate transaminase (AST) 69 U/l (normal range up to 31 U/l) and alanine transaminase (ALT) 50 U/l (normal range up to 34 U/l). Antinuclear antibody (ANA 1/160) was weakly positive while extractable nuclear antigens (ENAs) including anti-Jo-1 antibody were negative. Other laboratory parameters such as C-reactive protein (CRP) and lactate dehydrogenase (LDH) were normal. A histopathologic examination of the skin/muscle biopsy showed vacuolar degeneration of the basal membrane with perivascular inflammatory infiltration together with a lymphohistiocytic infiltration. In addition, an extensive mucin deposition in her dermis and linear atrophy of her muscle layer were detected. The morphologic features were compatible with dermatomyositis. An electromyogram and magnetic resonance imaging (MRI) of the muscles of her extremities showed a symmetric moderate myopathy mainly of proximal muscles which confirmed the diagnosis of dermatomyositis. A chest X-ray showed an unspecific pulmonary nodule in the upper field of her right lung. Computed tomography (CT) of her chest revealed a nodule of 20×22 mm in her right upper pulmonary field without mediastinal or axillar lymphadenopathy (Fig. 3). A CT-guided biopsy of the lung nodule revealed a lung adenocarcinoma of moderate differentiation. After diagnosis of the lung tumor was made, she underwent a thorough screening including CT of her abdomen and MRI of her head/neck in order to exclude other tumors, lymph metastases or organ metastases. Furthermore we performed an endoscopy of her upper digestive tract and a gynecological control to exclude any other types of cancer. All of them were negative. Her pulmonary function tests and echocardiography were normal as well.

She was given a presumed diagnosis of a lung adenocarcinoma with clinical manifestations of paraneoplastic dermatomyositis. No metastatic lesions were found, and there were no abdominal or cerebral abnormalities. A corticosteroid treatment with prednisolone 1 mg/kg/day was administered. In the absence of cardiologic or anesthesiologic contraindications she was assessed to be eligible for surgery. She underwent a right upper lobectomy. The histopathology showed a moderately differentiated adenocarcinoma that was pathologically staged as T1b N0 M0. In the postoperative period, she presented partial improvement of her skin lesions, muscle weakness and dysphagia.

## Discussion

Dermatomyositis is an idiopathic inflammatory disease which affects muscles and skin and is characterized by skin rash and inflammation. Skin manifestations include erythema over the face, neck and upper trunk known as “neckline V sign”, erythema on the extensor surface of extremity joints, erythematous papules located on the metacarpophalangeal and proximal interphalangeal joints, called “Gottron’s papules” or purple-red discoloration of the upper eyelids usually associated with eyelid edema “heliotrope sign”. Typical nail disorders include capillary telangiectasia along the distal nailfolds and periungual erythema. Photosensitivity and pruritus have also been described in patients with dermatomyositis. Creatine kinase elevation, abnormalities in an electromyogram and inflammatory lesions in skin and muscle biopsy are typical findings [2]. The hallmarks of classic dermatomyositis include skin manifestations, proximal muscle weakness and laboratory data showing muscle inflammation. The entity dermatomyositis sine myositis has already been described and is known as amyopathic dermatomyositis. It is thought to concern approximately 20 % of patients with dermatomyositis [3] and is associated with a risk for rapidly progressive fatal interstitial lung disease [4]. However, the entity dermatomyositis sine dermatitis has rarely been reported and little literature exists to support this variant [5].

Our patient was admitted to our clinic of dermatology for exclusion of contact allergy by her physician, because of an itching sensation involving her arms and legs. A thorough physical examination revealed light elevated erythematous papular lesions over her metacarpophalangeal and proximal interphalangeal joints and nail changes such as periungual erythema and capillary telangiectasia along the distal nailfolds on both hands, which were of no subjective clinical significance for our patient. Other typical clinical features of dermatomyositis such as heliotrope rash or erythema on the extensor surface of extremity joints were absent. Since respective clinical signs raised a suspicion of dermatomyositis we intensified our examination which additionally revealed pain and proximal muscle weakness, swallowing difficulties and an uncharacteristic weight loss of 10 kg in the last 3 months. As a sudden presentation of dermatomyositis may be of paraneoplastic origin we performed further testing in order to exclude malignancy.

An association between dermatomyositis and malignancy was described many years ago. Approximately 15 to 20 % of adult patients with dermatomyositis present with cancer at the time of the diagnosis or at some point during the follow-up [6]. Chow *et al*. [7] suggested that the risk of malignancy is higher in the first year after the diagnosis of dermatomyositis and reduces gradually over the following years. Chen *et al*. [8] showed that the risk of malignancy in patients with dermatomyositis is higher during the first 5 years after the diagnosis. Compared to the risk of malignancy in the general population, the increased risk of malignancy in patients with dermatomyositis remains higher through all years of follow-up [7, 8]. In a retrospective analysis of 618 patients with dermatomyositis, 198 (32 % of the patients) had cancer. In 115 cases (19 % of the patients) malignancy was found after the diagnosis of dermatomyositis. In 83 cases (13 % of the patients) dermatomyositis and cancer were diagnosed concomitantly. A similar correlation was shown by Fujita *et al*. [9] concerning lung cancer. Despite the fact that a majority of malignancies are diagnosed after dermatomyositis, the tumor may be diagnosed before or at the same time as the dermatomyositis, as in our case.

The most common types of cancer associated with paraneoplastic dermatomyositis are ovary, lung, pancreatic, stomach, colorectal cancer or non-Hodgkin lymphoma with a prevalence of 6 to 45 % of patients [10]. Among them, ovary cancer has the highest incidence worldwide [10, 11]. For all types of cancer, however, the risk of malignancy after a diagnosis of dermatomyositis is increased three times [10].

Although the pathogenetic mechanism of dermatomyositis related to underlying malignancy remains unclear, there are some hypotheses which suggest the manifestation of dermatomyositis as a result of an immune disorder, an immunologic response, a substance secretion by neoplastic cells or as a response to newly presented tumor-associated antigens. The symptoms of dermatomyositis vary in severity and number and usually precede the diagnosis of malignancy.

Sterzt reported a paraneoplastic dermatomyositis with coexisting stomach cancer for the first time in 1916 and Kankeleit described a correlation with breast cancer at the same time. Andreev [12] showed that dermatomyositis often coexists with lung cancer some years later, although only a few reports are available to confirm this statement.

Lung cancer can be divided into two categories: small cell carcinoma and non-small cell carcinoma with diverse clinical manifestations depending on the localization, the infiltration of co-structures, the metastases and the paraneoplastic manifestations. On histological examination, it can be classified in four categories: squamous cell carcinoma, adenocarcinoma, large cell carcinoma and small cell carcinoma [13]. Hypertrophic osteopathy has been described as the most common paraneoplastic syndrome associated with lung cancer [14].

There are data that suggest that the presence of dermatomyositis is a paraneoplastic manifestation of lung cancer. However, a coexistence with a lung adenocarcinoma is rather rare. Paraneoplastic dermatomyositis has a higher incidence in cases of small cell carcinoma, followed by squamous cell carcinoma and adenocarcinoma [9]. Among the four histological types of lung cancer, adenocarcinoma is the one most commonly found in non-tobacco smokers and women. It is characterized by its peripheral location and a worse prognosis caused by the early appearance of metastases [15].

Our patient, however, was a woman who was a cigarette smoker which differs from the abovementioned data given from the literature. She had an unusual manifestation of a lung adenocarcinoma with a mild dermatomyositis as a prodromal sign of her tumor disease. Fortunately, because of the early diagnosis of cancer, she had no metastases. Her paraneoplastic dermatomyositis presented in a rather mild form. Her papular lesions and nail disorders were the only typical signs in such a mild form and she was unaware of them. Other typical skin manifestations such as heliotropic rash and erythema of the upper trunk, neck and arms were absent. In this case report we showed that even if the complete clinical image of classic dermatomyositis is not present there is a high risk of an underlying malignancy.

Our patient additionally complained about persisting pruritus as her major symptom leading to her initial consultation with her physician before coming to our clinic. Although pruritus has already been described as a clinical feature of dermatomyositis it remains a low-yield symptom concerning the diagnosis and is rather underestimated [16]. Dundley *et al*. [17] showed that pruritus can severely affect the quality of life of patients with dermatomyositis. In our case report it is remarkable that our patient described pruritus as one of her major symptoms, when asked. Therefore, the management of pruritus may also be included in the therapeutical concept in dermatomyositis.

As the pathophysiology of paraneoplastic dermatomyositis remains unclear, no standard treatment regimen has been established yet. Therapy with high-dose corticosteroids is suggested as the first-line therapy. In patients with no therapeutical response to corticosteroids an immunosuppressive therapy with azathioprine, cyclosporine, mycophenolate or methotrexate may lead to an improvement of symptoms and better disease control. Therapy with human immunoglobulins administered intravenously (IVIG) is suggested as second-line therapy [18]. In any case, the control of the underlying malignancy is independent of the severity of dermatomyositis and remains the most important part of the therapy as dermatomyositis improves or resolves after cancer treatment. As patients with a tumor and dermatomyositis may have a worse prognosis and quality of life, early diagnosis and treatment of this paraneoplastic disease is highly important.

## Conclusions

Our case report illustrates that paraneoplastic dermatomyositis, even in a mild form without all of the typical skin manifestations, may appear as a prodromal sign of malignancy. As the prognosis of paraneoplastic dermatomyositis depends on the underlying malignancy, an early recognition and awareness of this entity is not only important for a prompt diagnosis of the tumor but also for a better prognosis and quality of life for these patients.

## Consent

Written informed consent was obtained from the patient for publication of this case report and accompanying images. A copy of the written consent is available for review by the Editor-in-Chief of this journal.
